# Supplementary material for: Effective Components and Molecular Mechanism of Agarwood Essential Oil Inhalation and the Sedative and Hypnotic Effects Based on GC-MS-Qtof and Molecular Docking
Source: Molecules. 2022 May 28;27(11):3483. doi: 10.3390/molecules27113483 (PMC9182217; doi:10.3390/molecules27113483)
Supplement: Supplementary file 1 [file molecules-27-03483-s001.zip › molecules-1714759-supplementary.pdf]

Supplementary materials: Establishment of HS-SPME-GC-QTOF analysis method and Molecular docking data

1. Fiber coating

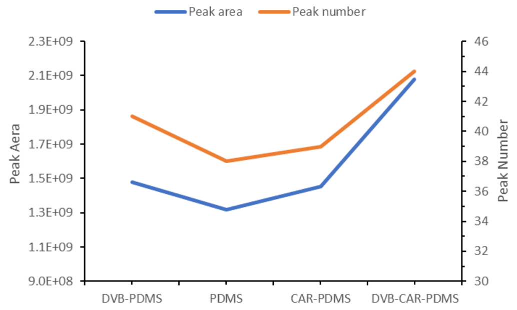

Figure S1. Effect of SPME fiber coating on the peak area and number of agarwood oil sample. (extraction temperature=40°C; extraction time=15min; desorption time=3min. The data were represented by the average of three replicate measurements.)

2. Extraction temperature

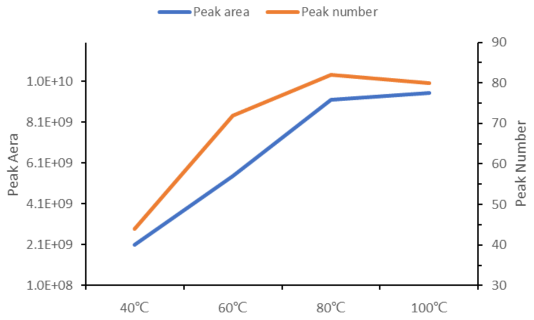

(a)

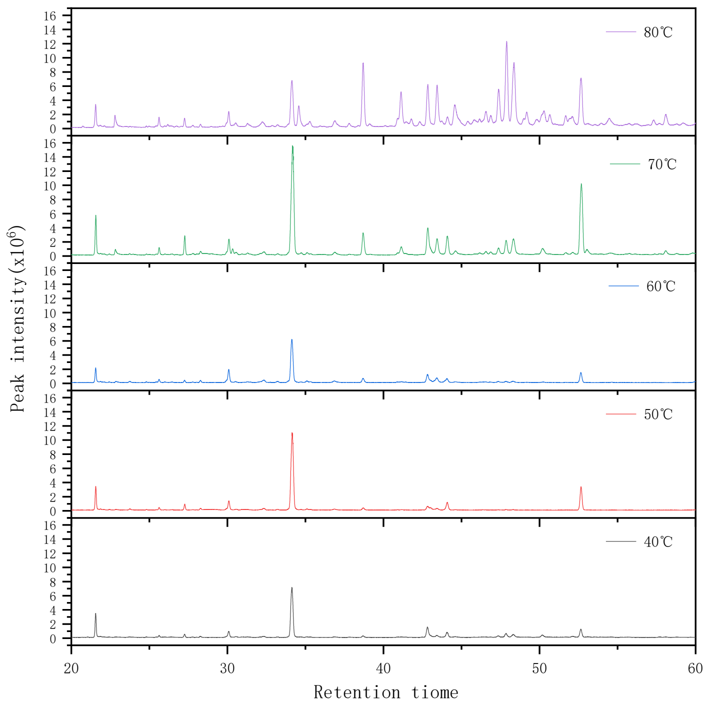

(b)

Figure S2. (a) Effect of extraction time on the peak area and number of agarwood oil sample by using DVB/CAR/PDMS fiber. (extraction time=15min; desorption time=3min. The data were represented by the average of three replicate measurements.) (**b**) The TIC chromatograph of different extraction temperature on serum sample by using DVB/PDMS fiber.

3. Extraction time

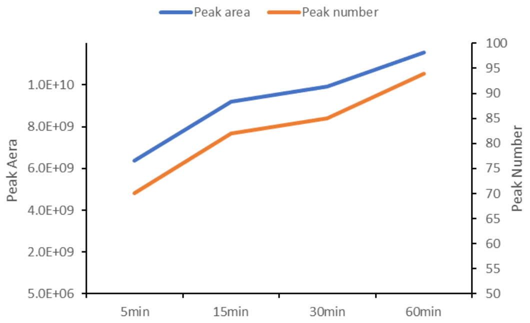

(a)

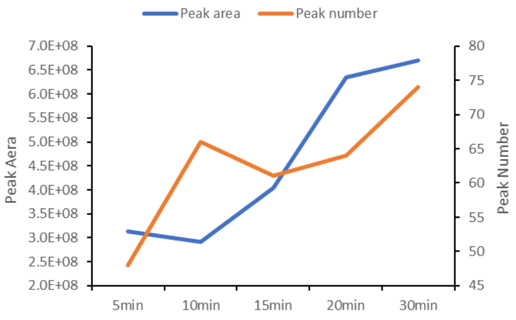

(b)

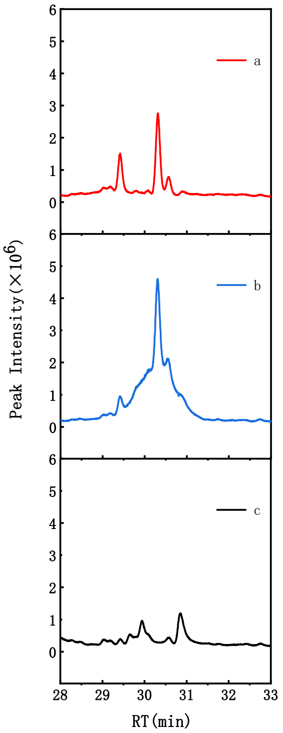

(c)

Figure S3. (**a**) Effect of extraction time on the peak area and number of agarwood oil sample by using DVB/CAR/PDMS fiber. (extraction temperature=80℃; desorption time=3min. The data were represented by the average of three replicate measurements.) (**b**) Effect of extraction time on the peak area and number of serum sample by using DVB/PDMS fiber. (extraction temperature=80℃; NaCl concentration=20mg. The data were represented by the average of two replicate measurements.) (**c**) Comparative TIC chromatograph analysis of different extraction time and salt concentration in RT29min-RT32min by using DVB/PDMS fiber.

(a: extraction time=20min, NaCl concentration=20mg; b: extraction time=20min, NaCl concentration=30mg; c: extraction time=30min, NaCl concentration=30mg;)

4. Salt concentration

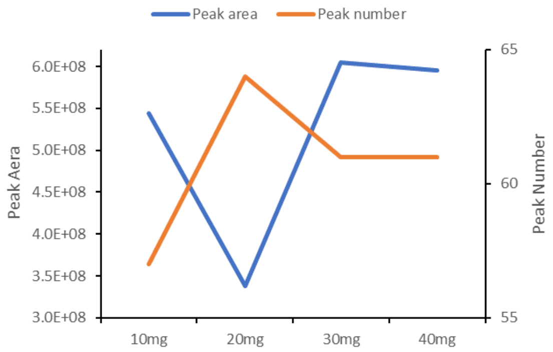

Figure S4. Effect of salt concentration on the peak area and number of serum sample by using DVB/PDMS fiber. (extraction temperature=80°C; extraction time=20min. The data were represented by the average of two replicate measurements.)

Table S1. Chemical composition of the 79 compounds putatively identified in agarwood oil sample.

Number	RT	Formula	Compound	Peak area/%	CAS	Similarity
1	7.169	C8H10O	Benzene, (methoxymethyl)-	0.16	538-86-3	76.53
2	7.938	C7H6O	Benzaldehyde	0.29	100-52-7	87.2
3	11.59	C10H16O	Carveol	1.43	99-48-9	75.54
4	12.075	C9H10O	Benzene, 1-ethenyl-4-methoxy-	0.07	637-69-4	84.1
5	16.047	C12H18	6,7-Dimethyl-1,2,3,5,8,8a-hexahydronaphthalene	0.1	107914-92-1	81.54
6	16.433	C10H12O	Benzenepropanal, .beta.-methyl-	2.43	16251-77-7	85.35
7	16.948	C10H12O	2-Butanone, 4-phenyl-	0.11	2550-26-7	84.73
8	18.921	C11H10	Naphthalene, 2-methyl-	0.24	91-57-6	71.57
9	19.845	C11H10	Naphthalene, 1-methyl-	0.11	90-12-0	80.92
10	21.425	C11H16O	4-(2-Methyl-cyclohex-1-enyl)-but-3-en-2-one	0.29	41437-92-7	79.26
11	22.114	C12H18O	2,7-Methanonaphthalene-3-methanol, 1,2,3,4,4a,7,8,8a-octahydro-	0.09	56045-33-1	80.62
12	24.529	C15H24	Bicyclo[5.2.0]nonane, 4-methylene-2,8,8-trimethyl-2-vinyl-	0.19	1000159-38-2	81.55
13(Z1)	24.835	C15H24	Bicyclo[5.2.0]nonane,2-methylene-4,8,8-trimethyl-4-vinyl-	0.68	242794-76-9	81.02
14(Z2)	25.656	C14H24O	Spiro[2.5]octane,5,5-dimethyl-4-(3-oxobutyl)-	1.72	77143-32-9	79.02
15	26.694	C15H22O	(1aR,4aS,8aS)-4a,8,8-Trimethyl-1,1a,4,4a,5,6,7,8-octahydrocyclopropa[d]naphthalene-2-carbaldehyde	0.08	470-41-7	79.55
16	27.13	C15H22O	Ylangenal	0.07	41610-68-8	80.72
17	27.785	C15H24	.alpha.-Guaiene	0.61	3691-12-1	87.15
18	28.058	C15H24	4,7-Methanoazulene, 1,2,3,4,5,6,7,8-octahydro-1,4,9,9-tetramethyl-, [1S-(1.alpha.,4.alpha.,7.alpha.)]-	0.34	514-51-2	84.92
19(Z3)	28.327	C15H24O	(1R,3aR,5aR,9aS)-1,4,4,7-Tetramethyl-1,2,3,3a,4,5a18,9-octahydrocyclopenta[c]benzofuran	1.54	104188-25-2	76.87
20	28.7	C15H24	(4S,4aR,6R)-4,4a-Dimethyl-6-(prop-1-en-2-yl)-1,2,3,4,4a,5,6,7-octahydronaphthalene	0.22	54868-40-5	85.01
21	28.972	C15H24O	(1R,4S,5S)-1,8-Dimethyl-4-(prop-1-en-2-yl)spiro[4.5]dec-7-ene	0.08	43219-80-3	83.99
22	29.268	C15H24O	Caryophyllene oxide	0.14	1139-30-6	82.24
23(Z4)	30.27	C15H24O	1H-Cycloprop[e]azulen-7-ol,decahydro-1,1,7-trimethyl-4-methylene-,[1aR-(1aα,4aα,7β,7aβ,7bα)]-	4.87	6750-60-3	89.51
24	30.363	C15H24O	Tricyclo[5.2.2.0(1,6)]undecan-3-ol, 2-methylene-6,8,8-trimethyl-	3.47	1000159-37-6	86.33
25(Z5)	30.592	C15H24	4a,8-Dimethyl-2-(prop-1-en-2-yl)-1,2,3,4,4a,5,6,7-octahydronaphthalene	1.24	103827-22-1	78.54
26	31.184	C15H24	.beta.-Guaiene	0.17	88-84-6	87.53
27(Z6)	31.39	C15H24	(1R,3aS,8aS)-7-Isopropyl-1,4-dimethyl-1,2,3,3a,6,8a-hexahydroazulene	2.2	36577-33-0	88.25

28(Z7)	32.468	C15H26O	2H-3,9a-Methano-1-benzoxepin,octahydro-2,2,5a,9-tetramethyl-,[3R(3α,5aα,9α,9aα)]-	4.92	5956-09-2	88.55
29(Z8)	33.29	C15H24O	(R)-2-((4aS,8aR)-4a-Methylene-1,4,4a,5,6,7,8,8a-octahydronaphthalen-2-yl)propan-1-ol	1.36	28102-68-3	74.9
30	34.051	C15H24O	2-(4a,8-Dimethyl-1,2,3,4,4a,5,6,7-octahydro-naphthalen-2-yl)-prop-2-en-1-ol	2.51	1000190-51-8	83.57
31(Z9)	35.235	C15H26O	1,4-Dimethyl-7-(prop-1-en-2-yl)decahydroazulen-4-ol	1.73	21698-41-9	76.36
32(Z10)	35.445	C15H26O2	Cedran-diol,(8S,14)-	1.38	62600-05-9	85.16
33	36.23	C24H40O2	10,12-Tricosadiynoic acid, methyl ester	0.38	1000333-59-4	81.06
34(Z11)	36.982	C15H24O	2-((2R,4aR,8aR)-4a,8-Dimethyl-1,2,3,4,4a,5,6,8a-octahydronaphthalen-2-yl)prop-2-en-1-ol	3.56	65018-15-7	89.11
35	38.066	C15H24	Bicyclo[5.3.0]decane, 2-methylene-5-(1-methylvinyl)-8-methyl-	0.93	1000159-39-3	90.35
36(Z12)	39.021	C15H26O	2-((4aS,8R,8aR)-4a,8-Dimethyl)-3,4,4a,5,6,7,8,8a-octahydronaphthalen-2-yl)propan-2-ol	6.31	194607-96-0	71.71
37(Z13)	40.547	C15H24O	2-((2R,4aR,8aS)-4a-Methyl-8-methylenedecahydronaphthalen-2-yl)prop-2-en-1-ol	0.15	515-20-8	81.27
38(Z14)	41.286	C15H24O	(E)-2-((8R,8aS)-8,8a-Dimethyl-3,4,6,7,8,8a-hexahydronaphthalen-2(1H)-ylidene)propan-1-ol	4.71	22387-74-2	75.9
39(Z15)	41.758	C15H24O	Aristol-1(10)-en-9-ol	3.39	1372763-27-3	79.1
40	42.397	C15H24O	Alloaromadendrene oxide-(1)	0.31		
41	42.656	C15H24O	7-Oxabicyclo[4.1.0]heptane, 2,2,6-trimethyl-1-(3-methyl-1,3-butadienyl)-5-methylene-	0.22	70038-20-9	79.73
42	43.072	C15H24O	.beta.-Oplopenone	0.28	28305-60-4	85.07
43	43.255	C23H34O2	4,7,10,13,16,19-Docosahexaenoic acid, methyl ester, (all-Z)-	0.11	2566-90-7	82.21
44(Z16)	43.614	C15H24O	Aromadendrene oxide-(2)	0.9	1000156-12-7	82.29
45	44.266	C15H24	.gamma.-HIMACHALENE	0.2	1000140-08-0	87.3
46(Z17)	44.858	C15H24	1H-Cyclopropa[a]naphthalene,decahydro-1,1,3a-trimethyl-7-methylene-,[1aS-(1aα,3aα,7aβ,7bα)]-	3.61	20071-49-2	89
47	45.041	C15H26O	1H-Benzocyclohepten-7-ol, 2,3,4,4a,5,6,7,8-octahydro-1,1,4a,7-tetramethyl-, cis-	1.4	6892-80-4	85.68
48	45.61	C15H24O	Cedren-13-ol, 8-	0.28	18319-35-2	83.49
49(Z18)	45.999	C15H24	1H-Cyclopropa[a]naphthalene,1a,2,3,3a,4,5,6,7b-octahydro-1,1,3a,7-tetramethyl-,[1aR-(1aα,3aα,7bα)]-	0.19	489-29-2	92
50(Z19)	46.378	C15H24	Guaia-1(10),11-diene	0.66	3691-11-0	93.79
51(Z20)	46.874	C15H24	β-Guaiene	1.76	88-84-6	85.53
52(Z21)	47.25	C15H24	(-)-Aristolene	1.8	6831-16-9	85.71

53(Z22)	47.712	C15H26O	1H-Cycloprop[e]azulen-4-ol,decahydro-1,1,4,7-tetramethyl-,[1aR-(1aα,4β,7α,7aβ,7bα)]-	1.48	552-02-3	86.63
54(Z23)	48.361	C15H24	Naphthalene,1,2,3,5,6,7,8,8a-octahydro-1,8a-dimethyl-7-(1-methylethenyl)-,[1S-(1α,7α,8aα)]-	4.06	10219-75-7	90.93
55	48.873	C15H24	Azulene, 1,2,3,3a,4,5,6,7-octahydro-1,4-dimethyl-7-(1-methylethenyl)-, [1R-(1.alpha.,3a.beta.,4.alpha.,7.beta.)]-	2.95	22567-17-5	92.06
56	48.906	C15H24	Longifolene	1.38	475-20-7	93.68
57	50.286	C15H22	3,5,11-Eudesmatriene	1.6	193615-07-5	86.42
58	50.516	C15H22O2	7-(1,3-Dimethylbuta-1,3-dienyl)-1,6,6-trimethyl-3,8-dioxatricyclo[5.1.0.0(2,4)]octane	1.58	1000190-22-7	84.99
59(Z24)	50.822	C15H24O	Tricyclo[4.4.0.0(2,7)]dec-8-ene-3-methanol,α,α,6,8-tetramethyl-,stereoisomer	2.33	41370-56-3	76.55
60	51.131	C15H24O	4a,5-Dimethyl-3-(prop-1-en-2-yl)-1,2,3,4,4a,5,6,7-octahydronaphthalen-1-ol	0.67	61847-19-6	89.41
61	52.239	C15H22O3	Hydroxyvalerenic acid	7.11	1619-16-5	83.34
62	52.718	C15H22O2	Curcumenol	0.11	19431-84-6	80.88
63	52.971	C15H22O	1(2H)-Naphthalenone, 3,4,4a,5,6,7-hexahydro-4a,5-dimethyl-3-(1-methylethenyl)-, [3S-(3.alpha.,4a.alpha.,5.alpha.)]-	0.7	562-23-2	84.88
64	53.506	C15H24O2	Bicyclo[4.4.0]dec-2-ene-4-ol, 2-methyl-9-(prop-1-en-3-ol-2-yl)-	0.64	1000196-79-7	84.67
65	54.604	C15H24O	(-)-Spathulenol	0.27	77171-55-2	82.96
66	55.891	C15H24O	(1R,7S,E)-7-Isopropyl-4,10-dimethylenecyclodec-5-enol	0.78	81968-62-9	84.5
67	56.403	C15H22O	(1aR,4aS,8aS)-4a,8,8-Trimethyl-1,1a,4,4a,5,6,7,8-octahydrocyclopropa[d]naphthalene-2-carbaldehyde	0.13	470-41-7	85.43
68	56.975	C15H22O	4,6,6-Trimethyl-2-(3-methylbuta-1,3-dienyl)-3-oxatricyclo[5.1.0.0(2,4)]octane	0.15	1000190-22-2	84.02
69	57.245	C15H22O	(E)-3-((4S,7R,7aR)-3,7-Dimethyl-2,4,5,6,7,7a-hexahydro-1H-inden-4-yl)-2-methylacrylaldehyde	0.29	4176-16-3	85.79
70	57.993	C15H22O	3-epi-Cedrenal	0.71	1000465-27-6	84.54
71	58.399	C15H22O	2H-Cyclopropa[a]naphthalen-2-one, 1,1a,4,5,6,7,7a,7b-octahydro-1,1,7,7a-tetramethyl-, (1a.alpha.,7.alpha.,7a.alpha.,7b.alpha.)-	2.87	6831-17-0	85.68
72	58.855	C15H22O	Squamulosone	0.32	34413-94-0	85.76
73	59.929	C15H22O	2-((2R,4aR,8aR)-4a,8-Dimethyl-1,2,3,4,4a,5,6,8a-octahydronaphthalen-2-yl)acrylaldehyde	0.8	4586-01-0	87.9
74	60.435	C15H24O	2-(4a,8-Dimethyl-1,2,3,4,4a,5,6,7-octahydro-naphthalen-2-yl)-prop-2-en-1-ol	0.13	1000190-51-8	81.67
75	60.85	C15H24O	2-((2R,4aR)-4a,8-Dimethyl-1,2,3,4,4a,5,6,7-octahy	0.1	65018-14-6	80.89

			dronaphthalen-2-yl)prop-2-en-1-ol			
76	62.367	C16H24O2	Methyl 2-((2R,4aR)-4a,8-dimethyl-1,2,3,4,4a,5,6,7-octahydronaphthalen-2-yl)acrylate	0.11	132342-55-3	90.54
77(Z25)	63.415	C15H22O	2(3H)-Naphthalenone,4,4a,5,6,7,8-hexahydro-4a,5-dimethyl-3-(1-methylethylidene)-,(4ar-cis)-	2.39	19598-45-9	90.66
78	63.987	C15H22O2	Valerenic acid	0.28	3569-10-6	86.26
79	69.109	C15H22O2	(3R,3aR,4aS,5R,9aS)-3,5,8-Trimethyl-3a,4,4a,5,6,7,9,9a-octahydroazuleno[6,5-b]furan-2(3H)-one	0.08	66873-38-9	87.15

Table S2. Docking results of twenty five components with four targets

Ligand	Receptor	affinity (kcal/mol)
Z1	GABRA1	-11
Z10	GABRA1	-11.6
Z11	GABRA1	-10.7
Z12	GABRA1	-10.9
Z13	GABRA1	-9.2
Z14	GABRA1	-10.2
Z15	GABRA1	-9.4
Z16	GABRA1	-11.1
Z17	GABRA1	-11.6
Z18	GABRA1	-11.5
Z19	GABRA1	-10.6
Z2	GABRA1	-9.5
Z20	GABRA1	-10.5
Z21	GABRA1	-11.8
Z22	GABRA1	-10.8
Z23	GABRA1	-10.7
Z24	GABRA1	-9.3
Z25	GABRA1	-9.4
Z3	GABRA1	-12
Z4	GABRA1	-11.6
Z5	GABRA1	-9.8
Z6	GABRA1	-10.5
Z7	GABRA1	-11.6
Z8	GABRA1	-10.7
Z9	GABRA1	-8.7
Z1	GRIA1	-10
Z10	GRIA1	-10.3
Z11	GRIA1	-9.7
Z12	GRIA1	-9.7
Z13	GRIA1	-9.7

Z14	GRIA1	-9.3
Z15	GRIA1	-8.9
Z16	GRIA1	-10.7
Z17	GRIA1	-10.6
Z18	GRIA1	-10.6
Z19	GRIA1	-9.6
Z2	GRIA1	-8.8
Z20	GRIA1	-9.6
Z21	GRIA1	-10.7
Z22	GRIA1	-9.9
Z23	GRIA1	-9.9
Z24	GRIA1	-10
Z25	GRIA1	-10
Z3	GRIA1	-10.5
Z4	GRIA1	-10.2
Z5	GRIA1	-10.2
Z6	GRIA1	-9.6
Z7	GRIA1	-10.7
Z8	GRIA1	-9.7
Z9	GRIA1	-9.2
Z1	HTR1A	-10.3
Z10	HTR1A	-10.7
Z11	HTR1A	-8.4
Z12	HTR1A	-10.6
Z13	HTR1A	-8.2
Z14	HTR1A	-9.8
Z15	HTR1A	-8.7
Z16	HTR1A	-10.9
Z17	HTR1A	-11
Z18	HTR1A	-11
Z19	HTR1A	-10.5
Z2	HTR1A	-8.8
Z20	HTR1A	-8.5
Z21	HTR1A	-11.2
Z22	HTR1A	-10.3
Z23	HTR1A	-10.1
Z24	HTR1A	-10.1
Z25	HTR1A	-10.4
Z3	HTR1A	-10.8
Z4	HTR1A	-10.9
Z5	HTR1A	-10.3
Z6	HTR1A	-10.5
Z7	HTR1A	-11.1

Z8	HTR1A	-7.7
Z9	HTR1A	-8
Z1	HTR2A	-9.5
Z10	HTR2A	-9.9
Z11	HTR2A	-9.3
Z12	HTR2A	-9.8
Z13	HTR2A	-9.3
Z14	HTR2A	-8.8
Z15	HTR2A	-8.4
Z16	HTR2A	-10.6
Z17	HTR2A	-10.3
Z18	HTR2A	-10.3
Z19	HTR2A	-9.2
Z2	HTR2A	-8.3
Z20	HTR2A	-9.2
Z21	HTR2A	-10.3
Z22	HTR2A	-9.6
Z23	HTR2A	-9.7
Z24	HTR2A	-9.1
Z25	HTR2A	-9.6
Z3	HTR2A	-10.7
Z4	HTR2A	-10.2
Z5	HTR2A	-9.8
Z6	HTR2A	-9.2
Z7	HTR2A	-10.7
Z8	HTR2A	-9.3
Z9	HTR2A	-9
